# Functional analysis of a novel, thyroglobulin-embedded microRNA gene deregulated in papillary thyroid carcinoma

**DOI:** 10.1038/s41598-017-10318-w

**Published:** 2017-08-30

**Authors:** Monika Kolanowska, Anna Wójcicka, Anna Kubiak, Michał Świerniak, Marta Kotlarek, Monika Maciąg, Paweł Gaj, Łukasz Koperski, Barbara Górnicka, Krystian Jażdżewski

**Affiliations:** 1 Genomic Medicine, Medical University of Warsaw, Warsaw, Poland; 2 Laboratory of Human Cancer Genetics, Centre of New Technologies, CENT, University of Warsaw, Warsaw, Poland; 3 Department of Pathology, Medical University of Warsaw, Warsaw, Poland

## Abstract

MicroRNAs, non-coding regulators of gene expression, are known culprits of thyroid cancer. Using next-generation sequencing, we identified a novel microRNA gene, encoded within an important thyroid regulator – thyroglobulin, and analyzed its functionality in the thyroid gland. *In vitro* and *in silico* analyses proved that the novel miR-TG is processed from the precursor, and co-expressed with thyroglobulin. Both genes are specific for thyroid tissue and downregulated in papillary thyroid carcinoma by 44% (p = 0.04) and 48% (p = 0.001), respectively. Putative target genes for miR-TG were identified using *in silico* tools, which pinpointed *MAP4K4*, an oncogene upregulated in thyroid cancer. Analysis of transcriptome by RNA-seq revealed that overexpression of miR-TG in PTC-derived cell line led to downregulation of several genes, including *MAP4K4* (fold change 0,82; p = 0.036). The finding was confirmed by SQ-PCR (fold change 071; p = 0.004). Direct interaction between miR-TG and *MAP4K4* was confirmed in the luciferase assay (p = 0.0006). Functional studies showed increase proliferation in K1 cell line transfected with miR-TG. We propose that in normal thyroid miR-TG plays a fine-tuning effect on the maintenance of MAPK pathway, inhibiting the expression of miR’s target *MAP4K4*. This regulation is disturbed in cancer due to downregulation of the novel, thyroglobulin-embedded microRNA, characterized in this study.

## Introduction

Papillary thyroid carcinoma (PTC) constitutes up to 85% of all thyroid cancer cases^1, 2^. It is generally acknowledged that most PTC cases exhibit constitutive activation of the Mitogen-Activated Protein Kinases/Extracellular signal-Regulated Kinases (MAPK/ERK) signalling pathway, in some cases due to mutations in *BRAF* and rearrangements of *RET/PTC* ^3^. Disturbed MAPK signalling underlies aberrant proliferation and apoptosis, leading to uncontrolled division and growth of affected cells. Importantly, most PTC cases do not exhibit RET/PTC rearrangements, or any other somatic mutations that could explain the observed alterations. A search for potential factors implicated in thyroid tumorigenesis led to conclusion that the genes culpable for the observed disturbances in MAPK signalling may not be of the traditional, protein-coding type, but include non-coding RNAs, such as microRNAs^4^.

MicroRNAs (miRNAs, miRs) are short (about 22 ribonucleotides long) non-coding RNAs. miRNAs are expressed as primary transcripts (pri-miRNAs) that are cleaved by the microprocessor complex to produce microRNA precursors (pre-miRNAs). Pre-miRNAs are transported to cytoplasm and transformed to mature miRNAs by the Dicer endonuclease. A mature miRNA is incorporated into the RNA induced silencing complex (RISC) and, subsequently, targets the 3′UTR (3′UnTranslated Region) of mRNA, leading to degradation of mRNA or inhibition of translation. The seed region of a miRNA, encompassing nucleotides 2–8 of the molecule is a basis for this pairing, and requires perfect complementarity with the target sequence^5^. Interestingly, although traditionally believed to be encoded in intergenic DNA, a significant number of miRNAs are embedded within the sequences of protein-coding genes. In such cases, miRNAs usually use the host gene’s transcription system^6^. This fact shed new light on the proper understanding of the cellular function of numerous genes, as their function might result from the concert action of both products of the gene, the protein and microRNA.

It is estimated that expression of more than a half of the protein-coding genes is regulated by miRNAs^7^, including oncogenes and tumor suppressors, and miRs play an important role in many processes such as apoptosis, cell proliferation, immune response and deregulation of hormones^8–10^. The expression of miRs is deregulated in many types of cancer. The implication of miRs in thyroid tumorigenesis has been widely studied^11–14^. He *et al*. detected preliminary evidence of the potential role for miRs in papillary thyroid carcinoma^11^ and other studies revealed that different microRNAs are deregulated in follicular and anaplastic thyroid carcinoma^12–14^, proving that each type of thyroid cancer harbors specific signatures of microRNA expression^15–17^. In our previous study, we employed next-generation sequencing to reveal the comprehensive microRNA profiles of the thyroid gland and PTC^18^, showing that 427 known microRNAs are expressed in thyroid tissue, and 44 of them are deregulated in cancer. However, to date no study aimed at identification of novel, previously unknown miRs expressed in the thyroid gland. In this study, we used the data obtained in next-generation sequencing^18^, with the aim to discover novel microRNAs embedded within important, protein-coding regulators of thyroid homeostasis. We identified a putative microRNA encoded within the thyroglobulin (*TG*) gene, whose protein product is one of the key factors involved in the biosynthesis of thyroid hormones. Interestingly, downregulation of thyroglobulin was frequently confirmed in thyroid tumors, including PTC^19, 20^. In further steps, we investigated maturation of the novel miR, provisionally referred to as miR-TG, and its interactions with putative target genes, encoding for members of the MAPK signaling pathway. Furthermore, the influence of the novel miR on the total transcriptome of thyroid cancer cells was analyzed, revealing its role in numerous important processes implicated in tumorigenesis.

## Results

### Identification of novel microRNAs

Mapping of the sequenced short RNAs to miRNA sequences deposited in miRBase as described in^18^ revealed that 40% of the obtained short RNAs did not align to any known microRNAs. These 18,484 reads were mapped to genes related to the thyroid physiology or cancer, and subjected to filtering of repetitive elements and other non-coding RNAs. Among the mapped reads, two putative microRNAs were localized in an intron of a *TG* gene at 8q24.22; 8:133133392–133133485. According to NGS analysis, levels of both short transcripts were downregulated in PTC. Mapping suggested that the reads constituted two putative microRNA molecules produced from both arms of the precursor sequence. The expression level of microRNA produced from the 5p arm was very low, reaching 0.036 RPM (reads per million). The expression level of microRNA produced from the 3p arm (provisionary named, miR-TG) was higher, reaching 5.7 RPM in PTC-N, 3.9 RPM in PTC-T, and 7 RPM in NN samples.

### The novel miRNA is encoded within the thyroglobulin gene

**Figure 1:**
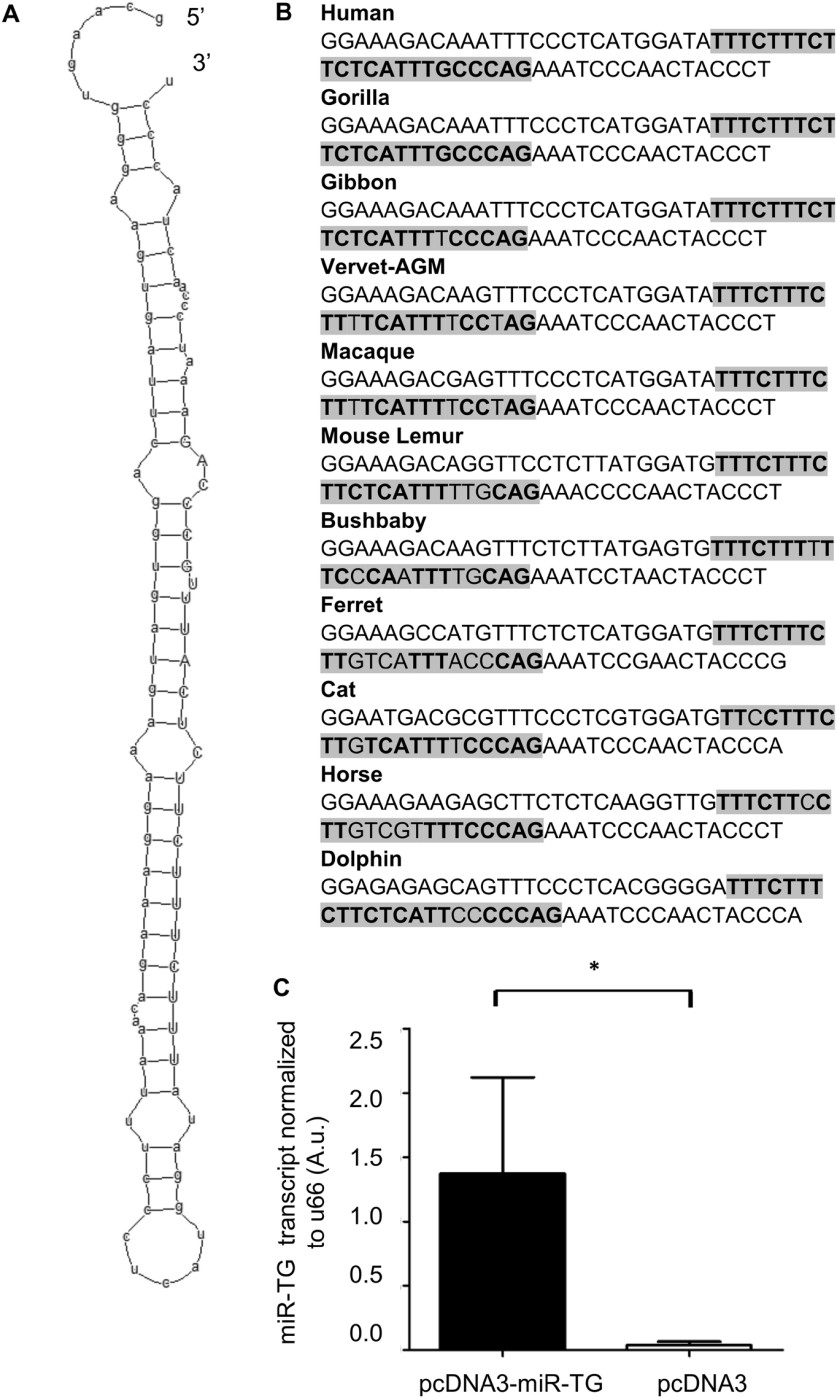
*In silico* and *in vitro* analysis of the precursor sequence for miR-TG. (**A**) Secondary structure of the pre-miRNA. The sequence of mature miRNA on the 3p arm of the hairpin is in upper cases. (**B**) Fragment of the evolutionary conserved sequence of pre-miRNA. The sequence of mature miR-TG is highlighted. (**C**) Relative expression of miR-TG normalized against *U66* was 45-fold upregulated in HeLa cells transfected with pcDNA3-miR-TG compared to cells transfected with pcDNA3 (control). The data are means ± S.E., unpaired t test; *p < 0.05.

*In silico* folding analysis revealed that the predicted microRNA precursor encoded within the *TG* gene formed a proper hairpin structure (minimum free energy (MFE) of −42.2, shape probability 84%)^21^, allowing for processing of mature miRNAs (Fig. 1A). Analysis of evolutional conservation revealed that the sequence of the putative miR-TG is present among many species of mammals, not only Primates (Fig. 1B), proving its evolutionary conservation. Thus, the performed *in silico* analyses indicated that the sequence potentially encoding for miR-TG could give rise to a functionally relevant microRNA precursor.

Transfection of HeLa cell lines with the pcDNA3-miR-TG plasmid showed that the precursor for miR-TG is expressed from the plasmid and cleaved by endogenous endonucleases to produce mature miR-TG, as revealed in an SQ-PCR assay performed with custom primers and probes detecting solely mature miR-TG molecules (Fig. 1C). The expression of miR-TG was fully detectable in cell lines transfected with miR-TG expression plasmid, while the miR-TG was not detected in cell lines transfected with the empty pcDNA3 plasmid.

**Figure 2:**
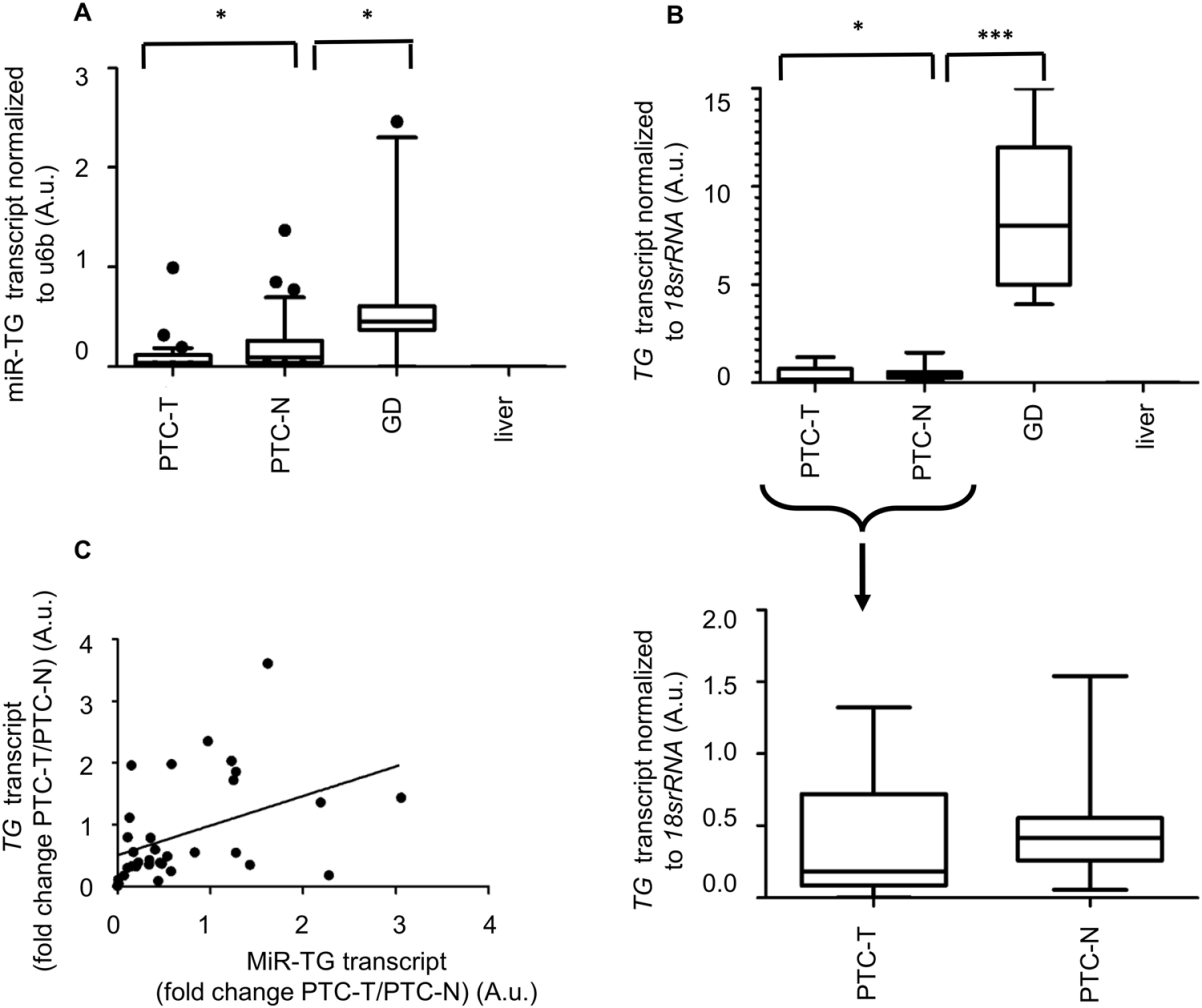
Expression of miR-TG and *TG* in tissue. Expression of miR-TG (**A**) and *TG* (**B**) normalized against *U6B* or 1*8srRNA* in tissue samples: PTC-T: PTC tumor, PTC-N-control tissue adjacent to tumor, GD-Graves’ Disease, liver-control liver tissue. Data are expressed as median and 10–90 percentile (miR-TG) or minimum-maximum range (*TG*). Statistical analysis was performed with a Mann-Whitney U test to compare expression of miR-TG and *TG* in PTC-N and different specimens, *p < 0.05, ***p < 0.0001. (**C**) Positive correlation between expression of the novel miRNA and *TG*, Spearman’s rank correlation.

The expression of miR-TG and *TG* is coupled and downregulated in PTC. SQ-PCR quantification of miR-TG in 33 pairs of PTC and adjacent, non-tumorous tissue revealed a 2.27-fold downregulation of the miR in PTC compared with control (p = 0.001, Fig. 2A). Thyroglobulin mRNA was measured in the same samples to reveal a 2.08-fold decrease (p = 0.04) in PTC compared with control tissue (Fig. 2B). The PTC-T/PTC-N fold change of expressions of both transcripts was positively correlated (r = 0.49, p = 0.003) (Fig. 2C).

The disease-associated changes in miR-TG expression were further evaluated in SQ-PCR analysis of tissue samples taken from patients suffering from Graves’ Disease (GD), a disorder associated with high levels of thyroglobulin. Additionally, liver specimens served as a control of tissue-specificity of miR-TG and *TG* expression (Fig. 2A,B). The expression of *TG* was 19-fold upregulated in GD compared with control thyroid tissue, PTC-N (p < 0.0001). Accordingly, the expression of miR-TG was 11.8-fold upregulated in GD compared with control thyroid tissue (p = 0.003). As expected, neither *TG* mRNA nor miR-TG were detectable in liver tissue, in which thyroglobulin is not expressed.

### *MAP4K4* is a target gene for the novel miR-TG

**Figure 3:**
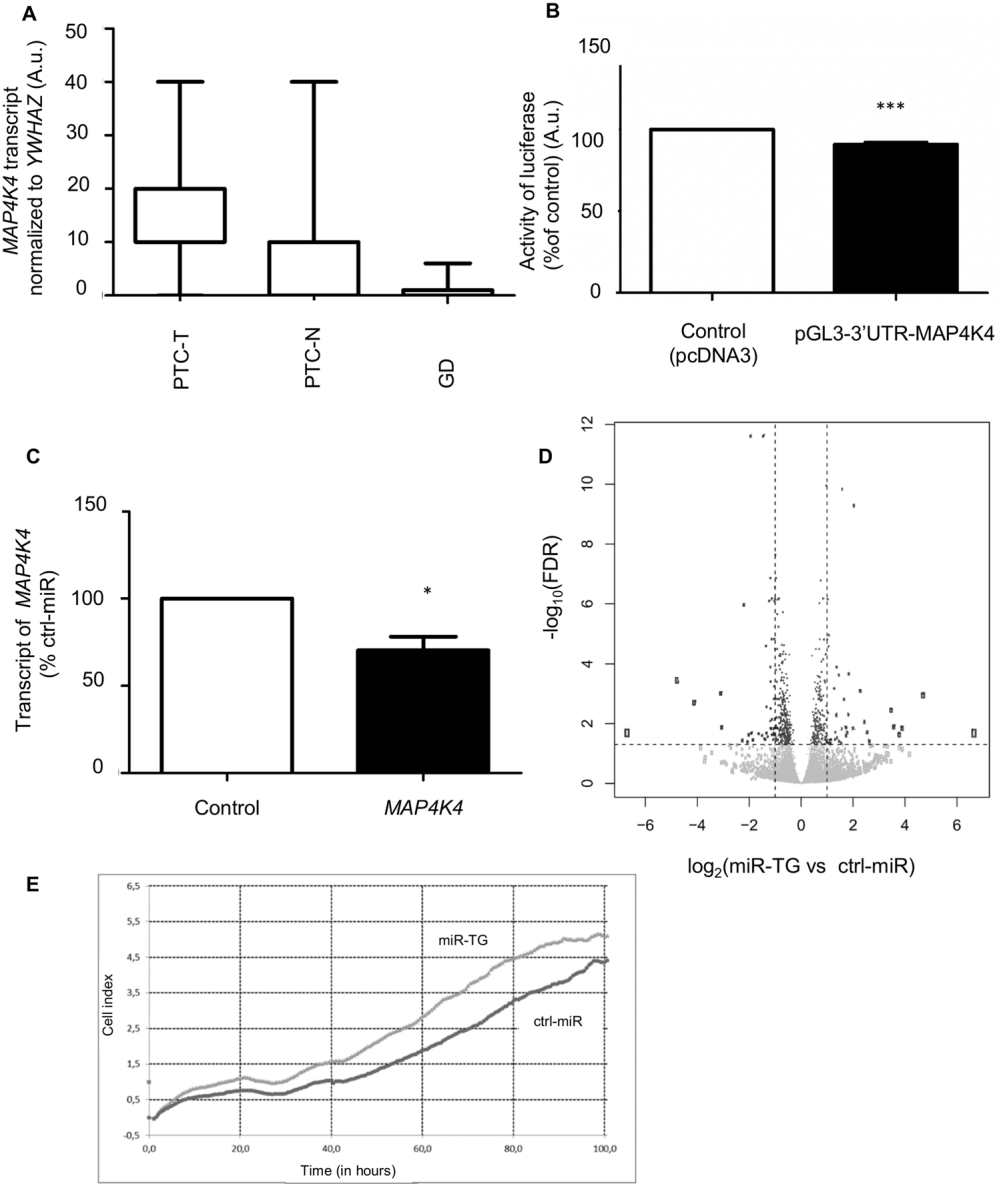
Functional analysis of miR-TG. (**A**) SQ-PCR of *MAP4K4* in tissue samples: PTC-T: PTC tumor, PTC-N-control tissue adjacent to tumor, GD-Graves’ Disease, normalized against *YWHAZ*. Mann-Whitney test was performed (p < 0,05), data expressed as median and maximum-minimum range. (**B**) HeLa cells transfected with pGL3-3′UTR-MAP4K4 and pRL-TK as an internal control. Firefly luciferase activity is showed as a percentage of control (cells transfected with pcDNA3 vector). The graph shows the mean, along with deviations from mean (SEM). Statistical analysis was performed using an unpaired t test ***p < 0.0001. (**C**) SQ-PCR of *MAP4K4* in K1 cell line transfected with pcDNA3-miR-TG or control. Results were normalized against *YWHAZ*. Data are expressed as mean values +/− SEM. Statistical analysis was performed using unpaired t-test to compare cells transfected with pcDNA3-miR-TG or control, *p < 0,05; (**D**) Comprehensive transcriptome of K1 cell line with miR-TG overexpression. Genes significantly upregulated and deregulated (FDR < 0.05) are marked respectively on left right side of graph. (**E**) The proliferation of K1 cell line transfected with pcDNA3-miR-TG or pcDNA3-ctrl-miR. The proliferation of K1 cells transfected with pcDNA3-miR-TG increases when compared to control. The experiment was ran on xCELLigence system (ACEA Biosciences, San Diego, California, USA) for 100 hours.

Putative target genes for miR-TG were identified using TargetRank and TargetScan *in silico* tools. We specifically focused on the genes with multiple binding sites for the miR, as well as the genes that were upregulated in thyroid cancer, and related to tumorigenic pathways. Using this approach, we selected MAPK pathway protein: *Mitogen Activated Kinase 4 Protein 4 (MAP4K4)*, which presents with multiple binding sites for miR-TG. We hypothesized that if *MAP4K4* was regulated by miR-TG, its mRNA levels should be negatively correlated with miR-TG expression. SQ-PCR revealed that the relative expression of *MAP4K4* was indeed highest in PTC and lowest in GD specimens: *MAP4K4* was 2.3-fold upregulated in PTC-T (p = 0.002), and 0.12-fold downregulated in GD (p = 0.04) (Fig. 3A).

Direct binding of miR-TG to the 3′UTRs of *MAP4K4* was confirmed in a luciferase assay. 3′UTR sequence of *MAP4K4* was cloned to the pGL3 control vector and the resulting plasmid was transfected to HeLa cell line, exhibiting low endogenous expression of both genes (data available upon request). Transfection of cells with pcDNA3-miR-TG and pGL3-3′UTR-MAP4K4 resulted in 9% (p = 0.0006) reduction of luciferase activity compared to control cells, transfected with an empty pcDNA3 vector (Fig. 3B).

### miR-TG downregulates expression of *MAP4K4* in PTC-derived cell lines

We employed K1 human thyroid cell line with low levels of miR-TG and relatively high level of *MAP4K4* to analyze the influence of miR-TG on endogenous expression of *MAP4K4*. Cells were transfected with pcDNA3-miR-TG or pcDNA3-ctrl-miR, expressing a control, mutated miR-TG. SQ-PCR analysis revealed a statistically significant decrease in *MAP4K4* (71%, p = 0.004) compared to control cells (Fig. 3C).

To further analyze the influence of miR-TG on the K1 cell line, we analyzed the complete transcriptome of cells transfected with miR-TG or control miRNA (Fig. 3D). Overexpression of miR-TG resulted in a significant (p < 0.05) deregulation of 2115 genes, including a significant downregulation of 1132 genes and upregulation of 983 genes (data available upon a request), including downregulation of *MAP4K4* (82%, p = 0.036). The analysis *TG* expression changes was not possible due to overlapping sequence of miR-TG. Furthermore, the performed Gene Ontology analysis revealed that deregulated genes are involved in pathways related to oxidoreductases activities and free reactive oxygen species (e.g. cytochrome c oxidase activity) and in protein translation mechanisms. Significantly altered pathways (p < 0.05) within the GO Biological Process, Molecular Function and Cellular Component are available upon request. The pathways are known as implicated in thyroid carcinogenesis.

Next we verified whether miR-TG impacts the MAP4K4 protein. The western blot analysis with measured densitometry of bands showed 9% decreased of MAP4K4 in cells transfected with pcDNA3-miR-TG when compared to control cells K1 transfected with pcDNA3-ctrl-miR, however the result was not significant (Figure S1).

### mir-TG affects proliferation of PTC - derived cell line

In order to verify whether miR-TG affects the proliferation of K1 cell line we employed xCELLigence system (ACEA Biosciences, San Diego, California, USA). The transfection of pcDNA3-miR-TG in K1 cells resulted in increase of cell index when compared to control (Fig. 3E).

## Discussion

Deregulation of microRNAs and a resulting aberrant expression of their target genes is a known factor implicated in the process of carcinogenesis. In this study, we discovered a novel, functional microRNA encoded within the thyroglobulin gene, whose downregulation in cancer leads to deregulation of pathways related to MAP kinase signaling. The novel miR-TG regulates a gene belonging to the MAPK signaling pathway, *MAP4K4*, what additionally strengthens its implication in thyroid carcinogenesis.

The first steps of identification of novel microRNA molecules require identification of their precursor sequences. The sequence of the novel miR was mapped in an intron of the thyroglobulin (*TG)* gene. According to known data miRNAs encoded within introns of host genes estimate over 30% of all human miRNAs^22^. *In silico* analysis of precursor sequence of miR-TG confirmed that it folds into a proper secondary hairpin structure with MFE: −42.2 kcal/mol with a shape probability of 84%, allowing for maturation of the miR. The methods of predicting secondary structures of pre-miRs are based on the selection of hairpin with the greatest number of paired nucleotides and, in consequence, the lowest MFE. Analysis of secondary structures of numerous pre-miRNAs showed that MFE is estimated at low values, ranging from −18 to −54 kcal/mol^23^. Since the putative precursor for miR-TG met this criterion, its proper folding and processing was highly probable. The sequence analysis and NGS results also showed that the predicted precursor encoded two mature microRNA molecules, what was a further proof of its biological validity. However, levels of miR-TG-5p in thyroid tissue were very low and thus the miR was not a subject of further study. With the use of pcDNA3-miR-TG expression plasmid we confirmed that miR-TG is proceeded to its mature form in a process typical for miRNA biogenesis.

Our study showed that miR-TG - similar to its host gene thyroglobulin - is specifically expressed in the thyroid tissue. Expression profiling showed that the expression of both miR-TG and thyroglobulin are downregulated in PTC. Deregulation of thyroglobulin in PTC was previously reported, however, the impact of this phenomenon on thyroid tumor progression remains unknown. The thyroglobulin is a precursor of thyroid hormones, but it is speculated that it also functions as a regulator of other genes, whose protein products are crucial for proper thyroid function^24^. Since miR-TG is encoded within the intron of *TG* it is possible that the regulatory role of thyroglobulin is in fact the contribution of miR-TG. According to published data the expression and function of miRNA and its host gene is very often coupled. By silencing of its target genes miRNA can support or inhibits the function of the protein product of its host gene^25, 26^.

Functionality of the miR-TG was further confirmed in experiments involving its putative target gene, *MAP4K4*, involved in MAPK pathways. Although sequences of 3′UTR of *MAP4K4* gene complementary to seed region of miR-TG were not evolutionary conserved, such interaction should still have a vast impact on potential gene silencing^27, 28^. *MAP4K4* it is the upstream activator of c-Jun N-terminal kinases (JNK), involved mainly in cell motility, rearrangements of cytoskeleton and cell proliferation^29, 30^. The overexpression of *MAP4K4* was noticed in many types of cancer^31^ including PTC^32, 33^. In this study we have comprehensively investigated the impact of miR-TG on *MAP4K4* expression in numerous experiments, including *in vitro* luciferase assay, *in vivo* gene expression analysis and NGS-based whole transcriptome profiling. We couldn’t confirm the effect of miR-TG on protein level of MAP4K4. However such result can be the effect of moderate regulatory role of miR-TG, which makes difficult to notice modest protein alteration in western blot analysis.

Overexpression of miR-TG in a PTC-derived K1 cell line led to alteration of numerous genes, including thioredoxin (*TXN*) involved in the redox homeostasis and protein synthesis. Increased oxidative stress, observed in thyroid tumors, is related to disorders of the redox system within cells^34^ including cytochrome c oxidase dysfunction^35^. Our results suggests that miR-TG is implicated in pathways related to the cytochrome c oxidase activity. In cell proliferation test we also revealed that miR-TG increases proliferation of K1 cell line.

In conclusion, our study suggests that the novel, thyroglobulin-embedded microRNA plays a fine-tuning effect on the maintenance of the thyroid gland homeostasis, and the downregulation of the miR-TG expression is involved in pathogenesis of PTC.

## Materials and Methods

### Ethics Statement

Tissue samples were collected with the approval of Institutional Review Board at Medical University of Warsaw and informed consents were obtained from the patients. All methods were performed in accordance with relevant guidelines and regulations.

### Thyroid tissue samples

For NGS experiment, samples of PTC (PTC-T, n = 14), matched control tissue from the same patient (PTC-N, n = 14) and non-cancerous thyroid samples (NN, n = 14) were collected with the approval of Institutional Review Board at the Medical University of Warsaw as described before^18^. For quantitative real-time PCR experiments, PTC-T (n = 33) and PTC-N (n = 33) samples were collected from 29 women and 4 men with median age of 51. Additional control samples from patients with Graves’ disease (GD) (n = 15), or liver tissue specimens (n = 9) were collected at the same Institution. Tumor staging was histopathologically confirmed based on the TNM classification of the World Health Organization (WHO). Total RNAs were extracted using TRiZol (Life Technologies, USA).

### Identification of novel miRNAs

Small RNA samples from PTC-T, PTC-N and NN were analyzed on Solid 5500 sequencing platform as previously described^18^. The obtained reads were subjected to quality control, and aligned to miRNA sequences deposited in miRBase ver 19^36, 37^. The remaining, unannotated sequences were analyzed by *silico* tools (NONCODE, Rfam, Repeat Masker, tRNAscan-SE) to filter out repetitive elements, tRNAs and other non-coding RNAs. The remaining reads, constituting putative novel microRNAs, were mapped to genes associated with the thyroid physiology or thyroid cancer: *BRAF, FOXE1, IGF1, NKX2-1, NTRK1, NTRK2, PAX8, RARA, RARB, RET, SLC26A4, SLC5A5, SLC5A8, TG, TGFB1, THRA, THRB, TP53, TPO, TSHR*.

### *In silico* analysis of the putative novel miRNAs

The structure of the putative novel miR-TG was analyzed using RNAshapes (Vienna RNA Package, version 1.5) and RNA structure 5.3 (University of Rochester Medical Center Mathews Lab) to assess if the putative pre-miRNA hairpin meets the criteria of minimum free energy (MFE) ≤ −30 kcal/ml and the shape probability ≥ 70%^21^. The analysis of evolutionary conservation of sequence was performed with the use of Enredo, Pecan, Ortheus (EPO) tools available on Ensembl Genome Browser.

TargetRank was used to predict target genes for the novel miR-TG. Only genes with both increased expression in thyroid tumors and potentially regulated by miR-TG were taken for further analysis.

### Plasmid construction

For miR-TG cloning, a 266 bp sequence encompassing the miR-TG precursor was amplified with the following primers: F-pre-miR-TG CCCGGTACCTCCCTCAGATACCGAGTGCAAGTG and R-pre-miR-TG CCCGGTACCGAGATGTACTTGGACCAGAAGGAGC using 100 ng of thyroid-derived genomic DNA under the following conditions: initial denaturation: 95 °C for 5 min; 35 cycles 94 °C, 20 s; 56 °C, 20 s; 72 °C, 15 s; final elongation 72 °C, 7 min, and cloned into the KpnI restriction site of pcDNA3 expression plasmid (Promega Corporation) to produce pcDNA3-miR-TG plasmid. For control, a synthetic nucleotide mimicking miR-TG with a mutated seed region (nucleotides 2–13) was amplified as above and cloned into the pcDNA3 plasmid to produce pcDNA3-ctrl-miR.

For luciferase assays, 3′UTR sequences of *MAP4K4* were amplified with primers: F-3′UTR-MAP4K4 CCCTCTAGAGCACAAGGAGGTGAGAAG, R-3′UTR-MAP4K4 CCCGGTACCGTGGGATGGGAAGCAAAC, using 100 ng of thyroid-derived genomic DNA under the following conditions: initial denaturation: 95 °C for 5 min; 35 cycles 94 °C, 20 s; 56 °C, 20 s; 72 °C, 150 s; final elongation 72 °C, 7 min, and cloned into the XbaI-KpnI restriction sites of pGL3-control vector (Promega Corporation) downstream the firefly luciferase gene.

### Transient transfection assays

For miRNA maturation analysis, HeLa cells (ATCC) were seeded at 10^5^/ml in a 12-well plate in DMEM (PAN Biotech) supplemented with 10% FBS (PAN Biotech, Germany) and transfected 24 hours later with 350 ng of the obtained pcDNA3-miR-TG or with 350 ng pcDNA3 (control) using Fugene HD (Promega Corporation). 24 hours after transfection, cells were collected, total RNAs extracted using the miRNA/RNA isolation kit (EurX, Poland) and the expression of mature miR-TG was analyzed in a quantitative real-time PCR.

For luciferase reporter assay, HeLa cells were seeded at 10^5^/ml in a 12-well plate in DMEM (PAN Biotech) supplemented with 10% FBS (PAN Biotech, Germany) and transfected 24 hours later using Fugene HD (Promega Corporation) with 500 ng of pGL3-MAP4K4 together with 170 ng of pcDNA3-miR-TG or empty pcDNA3. In all experiments, transfection with 100 ng of pRL-TK (Promega Corporation) served as an internal control. Luciferase activity was measured 24 hours after transfection in a Glomax-Multi Detection System (Promega Corporation) using Dual-Luciferase Reporter 1000 Assay System (cat.no. E1980, Promega Corporation). Data are represented as relative fold change of the ratios of firefly luciferase activity normalized to Renilla luciferase activity.

For endogenous gene expression analysis, K1 human cells (derived from papillary thyroid carcinoma, cat no. 92030501, ECACC) were seeded in 6-well plates in a 2:1:1 ratio medium mix containing DMEM, Hams F12 (PAN Biotech, Germany) and MDCB 105 (BIOWEST, France), supplemented with 10% FBS (PAN Biotech, Germany) and 4 mM Glutamine (PAN Biotech, Germany) and transfected 24 hours later with 1 µg pcDNA3-miR-TG or 1 µg pcDNA3-ctrl-miR using Fugene HD. Cells were collected and suspended in 500 ul of PBS 24 hours after the transfection and used for RNA and protein isolation.

### Quantitative real-time detection of miR-TG, *TG*, and *MAP4K4*

The RT-PCR primers: F-miR-TG CGCCGCTTTCTTTCTTCTCATT, R-miR-TG GTGCAGGGTCCGAGGT and probe: 6-FAM-TGGATACGACCTGGGC(MGB) for detection of miR-TG were designed according to Chen *et al*.^38^ and reaction conditions were adjusted using serial dilutions of a synthetic miR-TG mimic. *U66* (cat.no. 001002, Life Technologies) and *U6b* (cat.no. 001093, Life Technologies) served as reference gene for experiments performed in cell lines and tissue specimens, respectively. The reverse transcription reaction was performed using 80 ng of tissue-derived total RNA or 100 ng of cell line-derived total RNA for miR-TG, and 12 ng of total RNA or 100 ng for *TG or MAP4K4*, respectively, using random primers and M-MLV reverse transcriptase (Promega Corporation) in the following conditions: 16 °C-30 min, 42 °C-50 min, 85 °C-5 min. For real-time PCR, 1 µl of cDNA was amplified in the following conditions for miR-TG: initial denaturation: 95 °C-10 min followed by 50 cycles of 95 °C-10 s; 58 °C-30 s; 72 °C-1 s; for *TG*, *MAP4K4*: initial denaturation: 95 °C-10 min followed by 45 cycles of 95 °C-10 s; 58 °C-5 s; 72 °C-7 s. Due to differences in expression levels, *18srRNA* was used as reference gene for *TG*, and *YWHAZ* for *MAP4K4*. The primer sequences were: F-TG GAGGTTCCCTGAGGTATC, R-TG CAGGGTGGTTTCAGTGAAG for thyroglobulin, and F-MAP4K4 GCTAGAAGGCAGCAGGAACG, R-MAP4K4 CCTCTTCTAGCTGTCGCCTG for *MAP4K4* amplification.

### NGS analysis of transcriptome

Total RNAs were isolated from the K1 cell line transfected with pcDNA3-miR-TG or control plasmid. The quality of total RNA was confirmed on 2100 Bioanalyzer (Agilent, USA) with RNA 6000 Nano Kit (cat.no. 5065–4413, Agilent, USA) with RIN > 7. 3 µg of total RNA was used for library preparation according to TruSeq Stranded mRNA Library Prep Kit protocol (cat.no. RS-122-2101, Illumina, USA). Fragments of the length of 200–800 bp were extracted in a 3% agarose gel on BluePippin (Sage Science, USA) and sequenced using NextSeq. 500 High Output Kit-75 cycles (cat.no. FC-404-1005, Illumina, USA), on a NextSeq. 500 Instrument (Illumina, USA). Sequenced mRNAs were aligned to Human Genome GRCh38 available on Ensembl Genome Browser ver. 80.

### Western Blot

20 µg of protein of each sample was taken for electrophoresis. Proteins were transferred into the PVDF transfer membrane (Thermo). Membranes were incubated overnight in 10 °C with polyclonal anti MAP4K4 rabbit antibody – dilution in TBST 1:200 (AP8004a, Abgent), as a control protein we used β-actin antibody, dilution in TBST 1:3000 (GTX110564, GeneTex). After membrane was washed it was stained with secondary Goat Anti-Rabbit IgG Antibody (H + L) HRP conjugated, dilution in TBST 1:1500 (bs-0295G-HRP; Bioss). The membrane was washed and the protein bands were revealed in chemiluminesence reaction with use of Stella 8300, RayTest. The densitometry of bands was measured with use of Image J 1.45 s.

### Cell Proliferation assay

To analyze cell proliferation the xCELLigence system (ACEA Biosciences, San Diego, California, USA) was used. According to protocol K1 cell line was first plated on 6-well plates and transfected with 2 µg pcDNA3-miR-TG or 2 µg pcDNA3-ctrl-miR and then after 12 hours cells were transferred to 16 well plate for further analysis of cell proliferation. The analysis of proliferation was based on the electrical resistance of cells that adhere to electrodes on the surface measured for 100 hours.

### Statistical Analysis

Normally distributed data were analyzed using the Student’s t-test, non-normally distributed data were analyzed using Wilcoxon and Mann–Whitney U tests and correlation analysis was performed using the Spearman’s rank correlation coefficient (r). Statistical analysis was performed by R i386 3.0.1 software and GraphPad Prism 5. For NGS analysis, significantly deregulated mRNAs were identified using a moderated paired t -test allowing for pair effects in a linear model. Deregulated pathways were analyzed using the Gene ontology (GO) analysis.

## Electronic supplementary material

Figure S1
