# Supplementary material for: Functional analysis of a novel, thyroglobulin-embedded microRNA gene deregulated in papillary thyroid carcinoma
Source: Sci Rep. 2017 Aug 30;7:9942. doi: 10.1038/s41598-017-10318-w (PMC5577171; doi:10.1038/s41598-017-10318-w)
Supplement: Supplementary file 1 — Figure S1 [file 41598_2017_10318_MOESM1_ESM.pdf]

Supplementary Information

Functional analysis of a novel, thyroglobulin-embedded microRNA gene deregulated in papillary thyroid carcinoma.

Monika Kolanowska[1,2], Anna Wójcicka[1,2], Anna Kubiak[1,2], Michał Świerniak[1,2], Marta Kotlarek[1,2], Monika Maciąg[1], Paweł Gaj[2], Łukasz Koperski[3], Barbara Górnicka[3] and Krystian Jażdżewski[1,2*]

(1) Genomic Medicine, Medical University of Warsaw, Warsaw, Poland,
(2) Laboratory of Human Cancer Genetics, Centre of New Technologies, CENT, University of Warsaw, Warsaw, Poland
(3) Department of Pathology, Medical University of Warsaw, Warsaw, Poland

[*] Corresponding author: Krystian Jażdżewski, kjazdzewski@wum.edu.pl

A

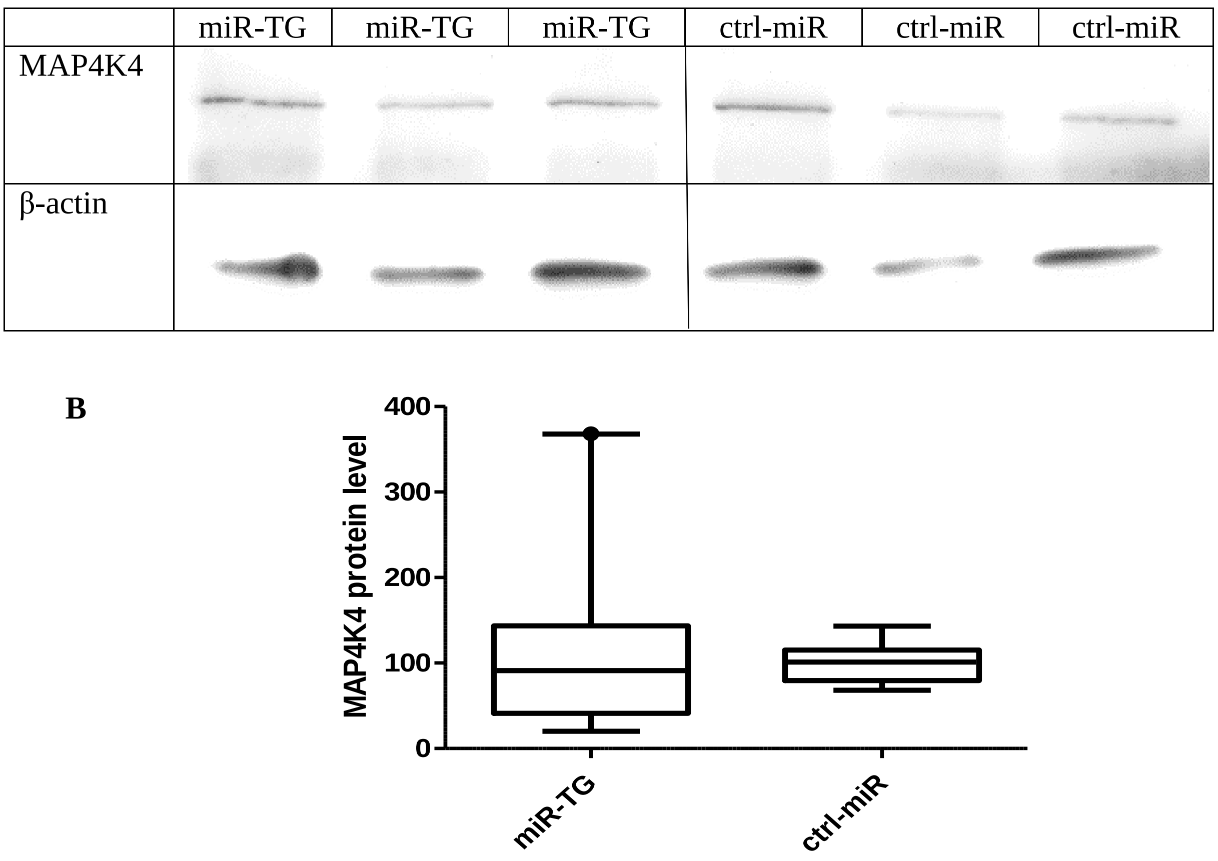

Figure S. 1 (A) Western blot analysis of MAP4K4 protein in K1 cell line transfected with pcDNA3-miR-TG or pcDNA3-ctrl-miR. β-actin was used as the reference protein. (B) The densitometry of western blots showed no significant decrease of MAP4K4 protein in K1 cells transfected with pcDNA3-miR-TG, when compared to control transfection with pcDNA3-ctrl-miR.
